# Secular trend analysis of antibiotic utilisation in some hospitals in Southern Sichuan from 2010 to 2020

**DOI:** 10.1038/s41598-023-35138-z

**Published:** 2023-05-19

**Authors:** Zhigui Wu, Yuan Li, Rong Li, Xuemei Sun, Tao Chen, Yongqi Yu, Yue Shi, Hongli Luo

**Affiliations:** 1 Department of Pharmacy, Affiliated Hospital of Southwest Medical University, Luzhou, 646000 China; 2 Department of Pharmacy, Gulin County People’s Hospital, Luzhou, 646000 China; 3 Department of Pharmacy, Luzhou People’s Hospital, Luzhou, 646000 China

**Keywords:** Diseases, Health care, Medical research

## Abstract

In order to assess the effectiveness of the Special Antimicrobial Stewardship Campaign launched by Ministry of Health of China in 2011, this study focused on the effectiveness and trends in the clinical use of antimicrobial drugs in selected hospitals in Southern Sichuan, China. This study collected and analyzed antibiotic data from 9 hospitals in Southern Sichuanin 2010, 2015, and 2020, including the rate of antibiotic use, expense, the intensity of antibiotic use and antibiotic use during the type I incisions of perioperative period. After 10 years of continuous improvement, the utilization rate of antibiotics in outpatients of the 9 hospitals continued to decline and was controlled below 20% by 2020, while the utilization rate in inpatients also significantly decreased, most were controlled within 60%. The use intensity of antibiotics (DDD (defined daily doses) per 100 bed-days) decreased from an average of 79.95 in 2010 to 37.96 in 2020. The prophylactic use of antibiotics decreased significantly in type I incision. The proportion of use within 30 min–1 h before operation was significantly increased. After the special rectification and sustained development of the clinical application of antibiotics, the relevant indicators of antibiotics tend to be stable, indicating that this Administration of antimicrobial drugs is conducive to improving the level of rational clinical application of antibiotics.

## Introduction

As early as 2000, the World Health Organization (WHO) raised concerns about antimicrobial resistance, which is a whole-of-society problem^1^. If governments around the world can’t control infection and slow down the development of drug resistance, human beings may die of various infectious diseases caused by superbug without effective treatment^2^. Resistance to multiple antimicrobial drugs occurs in different types of bacteria, for example, for bacteria with broad-spectrum lactamases can be treated with third or fourth generation cephalosporins, while for ESBL-like bacterium are resistant to third or fourth generation cephalosporins, enzyme inhibitors can target their resistance, while for AMPC-like bacterium are resistant to enzyme inhibitors, carbapenems may be a better choice^4^. In the case of abusing antibiotics, it is easy to produce extensive drug resistance. It is a very dangerous super bacterium, which has sounded an alarm to mankind^5^. The rational use of antibiotics is a major issue related to human health. Antibiotics are a double-edged sword. Rational use antibiotics can effectively treat infectious diseases caused by pathogenic bacteria, and greatly reduce deaths caused by infection. However, unreasonable use antibiotics can lead to the continuous growth of bacterial drug resistance, increase heavy resistance to the cure of diseases, and then affect the health of all mankind^6^.

How to reasonably control the use of antibiotics and reduce the production of drug-resistant strains has become a matter of widespread concern all over the world^7^. It is reported that if relevant measures are not taken, about 300 million people in the world will die prematurely (10 million per year by 2050), and economic losses will amount to $10 trillion^8^. Therefore, maintaining the effective effect of antibiotics and limiting the microbial drug resistance should be the common goal of each country. Nosocomial infection surveillance in Japan was antibiotic resistance surveillance, and was shared with the WHO global antibiotic resistance monitoring system, which provides a favorable tool for the monitoring of drug-resistant bacteria^9^. The United States points out that overuse of antibiotics is a major health care problem, leading to antibiotic resistance, including unnecessary long-term antibiotic treatment for patients with common bacterial infections, such as acute bronchitis with exacerbation of chronic obstructive pulmonary disease (COPD), community-acquired pneumonia (CAP), urinary tract infection (UTIs) and cellulitis (these infections require appropriate prescription and short-term antibiotic treatment)^10^. Canada has also took action on antimicrobial resistance and antimicrobial use^11^. This will increase the effectiveness of treatment of infectious diseases and facilitate compliance with international guidelines on antibiotic resistance^12^. China is one of the largest antibiotic consuming countries in the world, and faces the threat of antibiotic resistance^13^. In order to further strengthen the clinical application management of antibiotics in medical institutions, promote the rational use of antibiotics, effectively control bacterial resistance and ensure medical quality and safety, China has taken a number of measures to strengthen the management of antibiotics^14^. The Chinese government has been committed to solving the problem of improper use of antibiotics by strengthening the management of antibiotics^15^. In 2011, *National Health and Family Planning Commission* (NHFPC) launched a Special Antimicrobial Stewardship Campaign (SAC) to strengthen the management of antibiotic clinical use in all secondary (with 500 patient beds or fewer) and tertiary (with > 500 patient beds) hospitals in China^16^. The scholars^16^ are exploring the results achieved after the special rectification of antibiotics in China, such as analyzing the use of antibiotics through procurement data of antibiotics, For example, total hospital antibiotic use in China increased from 4.8 DID in 2010 to 6.7 DID in 2018, but this was different across provinces. It also varies by hospital level, with population-weighted antibiotic use higher in level II hospitals than in level III hospitals (7.3 DID vs. 6.6 DID).

Although there are good policy documents to support it, we need to monitor use of antibiotic over the past 10 years, especially in Western China, which is a sub developing region in China. Therefore, we studied and analyzed the use and change trend of antibiotics in 2010 (before special rectification of antibiotics), 2015 (during the special rectification of antibiotics) and 2020 (after the special rectification of antibiotics) in 9 hospitals in Southern Sichuan, so as to provide reference for further improving the monitoring and management of clinical application of antibiotics and promoting the rational use of antibiotics in medical institution.

## Methods

### Study design

This study was a retrospective observation of antibiotic use in different hospitals in Southern Sichuan from 2010 to 2020. NHFPC has been leading the antibiotic management campaign for 10 years. Clinical pharmacists in each hospital carry out a variety of interventions such as antimicrobial prescribing reviews, medical advice audits and antimicrobial use cultivation and performance management.

According to the hospital directory of the southern Sichuan region, we selected the best public hospitals in different counties or cities, not containing specialist hospitals, and having a hospital network information system capable of improving data. Finally, nine hospitals were selected for the survey.

We designed questionnaires and sent to the Pharmacy Management and Pharmacotherapeutics Committee (PMPC) of selected 9 hospitals. These questionnaires are The contents of questionnaires include the drug name, drug specification, dosage form, consumption quantity, drug cost, utilization rate, use intensity, perioperative use of antibiotics for type I incisionsin 2010, 2015 and 2020, covering the situation before, during and after the SAC in 2011. For confidentialy reasons, deidentified information regarding hospitals were collected.

### Data collection and management

In this study, the main indicators and calculation methods are as follows:

1 $$\mathrm{Usage rate of antibiotics in outpatients}=\frac{\mathrm{number of antibiotics used in outpatients}}{\mathrm{total number of outpatients}}*100\%$$

2 $$\mathrm{Usage rate of antibiotics in inpatients}=\frac{\mathrm{total number of discharged patients using antibiotics}}{\mathrm{total number of discharged patients}}*100\%$$

3 $$\mathrm{Intensity of antibiotics use per }100\mathrm{ bed}-\mathrm{days}=\frac{\mathrm{consumption of antibiotics in inpatients }(\mathrm{cumulative DDDs})}{\mathrm{number of days of patients admitted during the same period }}*100$$

Note: Number of patients during the12 months = number of patients during the12 months * average length of stay of patients during the12 months.

The DDD of the drug follows the recommendations of the WHO Collaborating Center for drug statistical methods. For those that cannot be queried, the administration scheme recommended in the manufacturer's instructions approved by the State Food and Drug Administration shall be followed. Drugs with the same chemical name and different specifications are counted as the same kind of drug, and the same kind of drug with different routes of administration are counted separately^17^.

4 $$\mathrm{Percentage of antimicrobial drug cost}=\frac{\mathrm{Total cost of antimicrobial drugs}}{\mathrm{Total cost of all drugs}}*100\%$$

Note: The unit of drug cost is RMB (¥, Ren min bi)

5 $$\mathrm{Utilization rate of antibiotics used in type I incision surgeries}=\frac{\mathrm{umber of prophylactic antibiotics used in type I incision surgeries}}{\mathrm{total number of type I incision surgeries}}*100\%$$

6 $$\mathrm{Rational rate of antibiotics in type I incision operation within }0.5\mathrm{ to }1\mathrm{ h before operation}=\frac{\mathrm{number of patients administered within }0.5\mathrm{ to }1.0\mathrm{ h before type I incision surgery}}{\mathrm{ttotal number of patients administered for type I incision surgery}}*100\%$$

7 $$\mathrm{Percentage of prophylactic drugs less than }24\mathrm{ hours in type I incision surgery}=\frac{\mathrm{number of antibiotics used in outpatientsnumber of prophylactic drugs less than }24\mathrm{ hours in type I incision surgery}}{/\mathrm{total number of prophylactic drugs in type I incision surgery }}*100\%$$

According to the Guidelines for the Clinical Use of Antimicrobial Drugs, the indicators related to the rational use of antimicrobial drugs are as follows: the number of antibiotics procured in secondary and tertiary hospitals is limited to 35 and 50 respectively; Usage rate of antibiotics should be distributed to less than 60% and 20% in inpatient and outpatient settings; the intensity of antibiotic use should be less than 40 daily defined doses (DDD) per 100 bed-days; Utilization rate of antibiotics used in type I incision surgeries should be reduced to 30%.

## Results

### Basic information of the 9 hospitals

**Figure 1:**
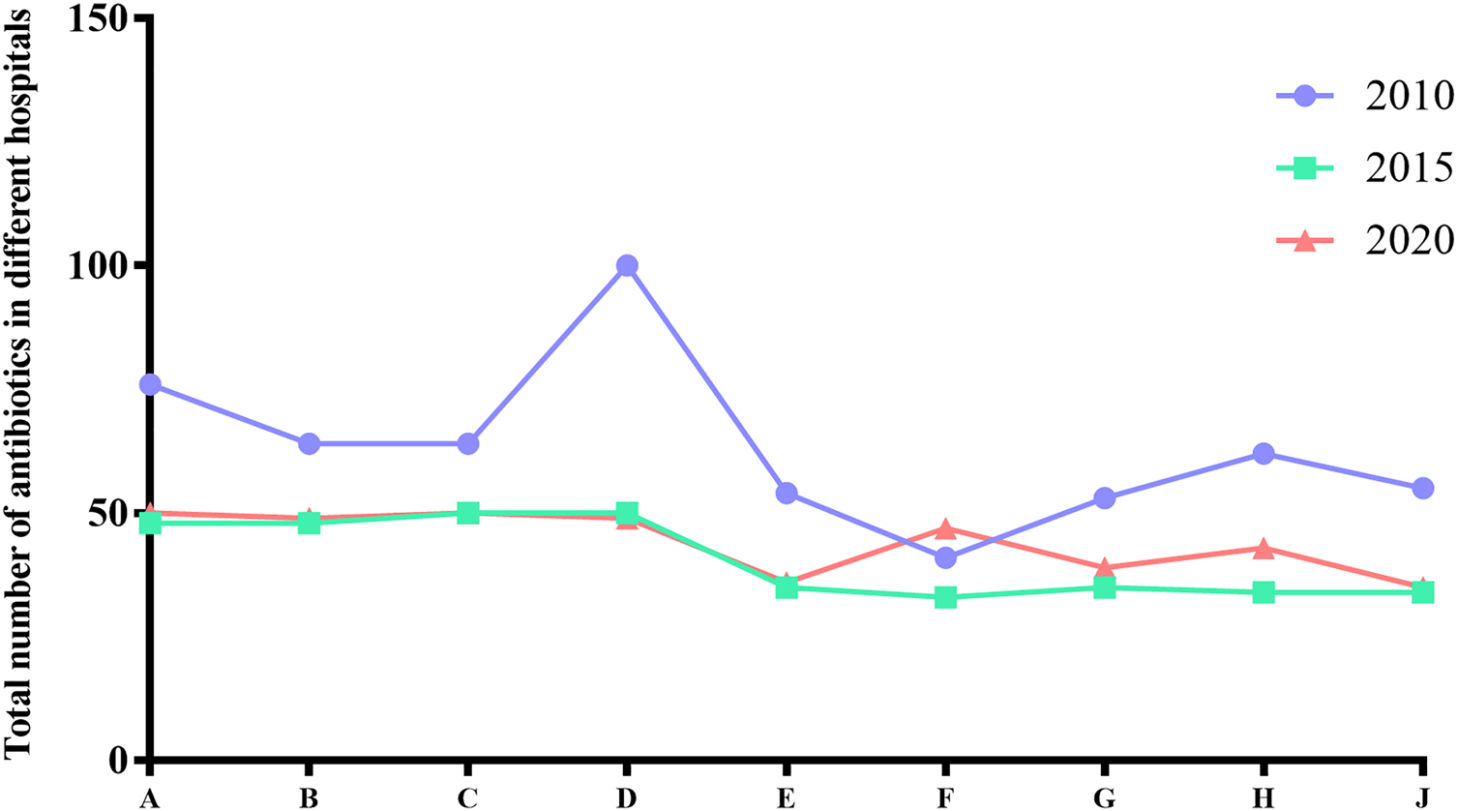
Total number of antibiotics in different hospitals in 2010, 2015 and 2020.

The basic information about the selected 9 hospitals, such as outpatient visits, the number of inpatients, Average length of stay for hospitalized patients (days), the total hospital drug cost (million), the antibacterial drug cost, and ratio of antibacterial drug cost to total drug cost (Fig. 1).

In 2010, only hospital A and hospital B were tertiary hospitals, while others were secondary hospitals. In particular, hospital A is a comprehensive hospital integrating medical treatment, teaching and scientific research, so its outpatient visits and inpatients are much higher than those of other hospitals. After 10 years of development, the number of outpatient visits in each hospital, the number of hospitalized patients and the drug expense of the whole hospital increased significantly. The average length of stay in most hospitals has decreased (Fig. 1c). Taking hospital A as an example, the number of outpatient visits increased by 160.6%, the number of inpatients increased by 87.0%, and the drug cost of the whole hospital increased by 143.6%, but the proportion of the expense of antibiotics in the total expense of drugs in the whole hospital decreasing. According to the SCA, the percentage of antimicrobial drug use is controlled to less than 20%. It showed that the overall control of antibiotics was acceptable.

### The variety of antibiotics in selected 9 hospitals

**Figure 2:**
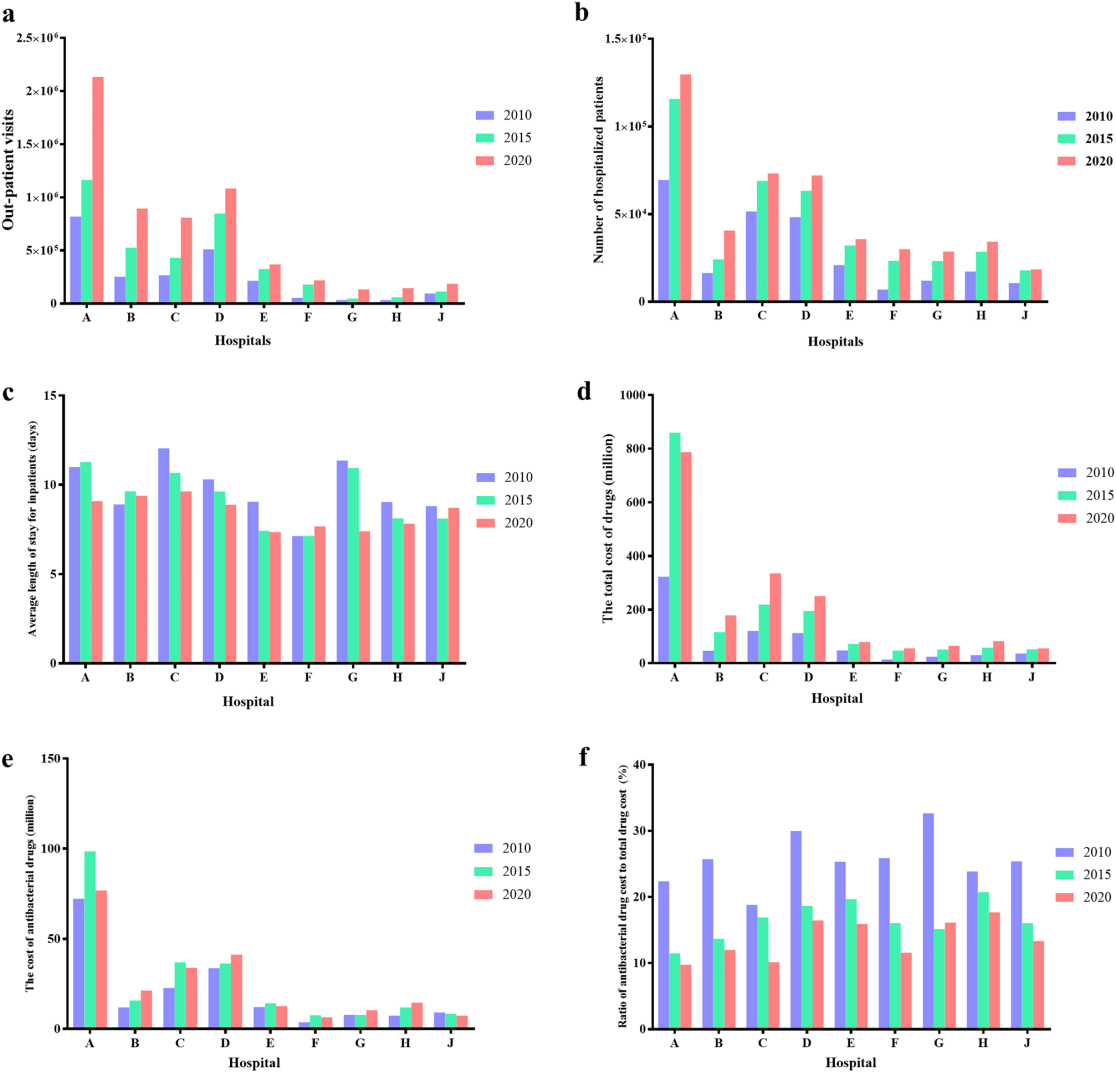
Basic information of the nine hospitals in 2010, 2015 and 2020. (**a**) The total number of outpatient visits; (**b**) The total number of hospitalized patients; (**c**) Average length of stay of hospitalized patients; (**d**) Total drug cost of the hospital; (**e**) Total cost of antibacterial drugs in the hospital; (**f**) Ratio of antibacterial drug cost to total drug cost in the hospital.

**Figure 3:**
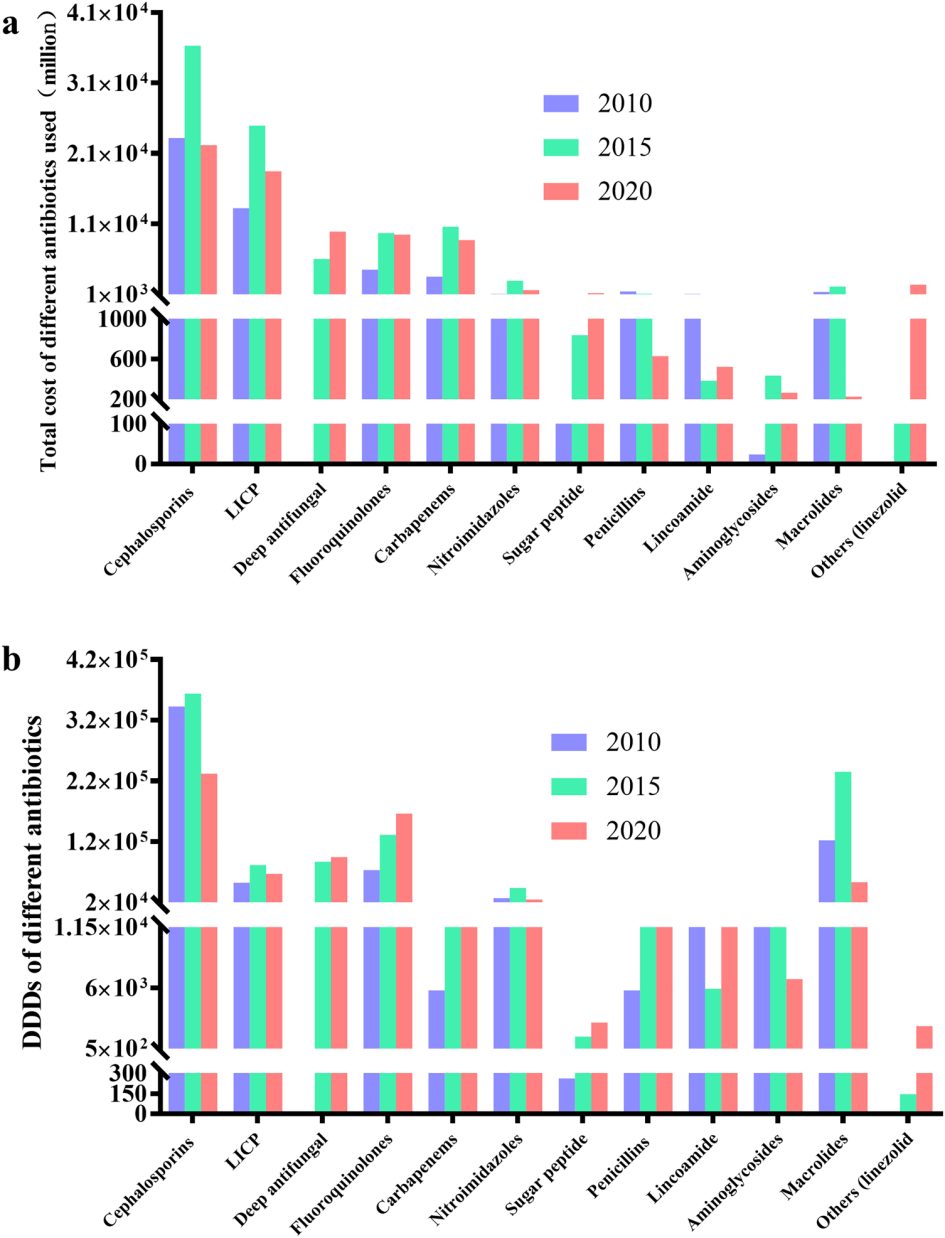
Usage of different antibiotics in hospital A. (**a**) Total drug cost of different antibiotics used in hospital A; (**b**) DDDs of different antibiotics in hospital A. LICP: lactamase inhibitor compound preparation.

The use of antibiotics in the hospital can be preliminary understood from the number of antimicrobial drug ATC categories in the hospital. According to Fig. 2, the number of antimicrobial drug ATC categories in 2010 in both secondary and tertiary hospitals exceeded the target values required by the SAC. But, in 2015 and 2020, the number of reached the requirements (No more than 50 for tertiary hospitals, and no more than 35 in the secondary hospitals) the specific categories and use of antimicrobial drugs were illustrated in the hospital A. The main varieties include penicillins (without β*-lactamase*), cephalosporins (without enzyme inhibitors), carbapenems, β lactamase inhibitor compound preparation (β-LICP), macrolides, lincosamides, fluoroquinolones, aminoglycosides, nitroimidazoles, glycopeptides, etc. (Fig. 3). The revenueof cephalosporin used was much higher than other antibacterial drugs in 2010, 2015 and 2020, followed by β lactamase inhibitor compound. The revenue of penicillins decreased while that of glycopeptides increased. In terms of DDDs of antibiotics, cephalosporins were the highest, macrolides and quinolones followed (Fig. 3).

### Use of antibiotics in outpatients of 9 hospitals

**Figure 4:**
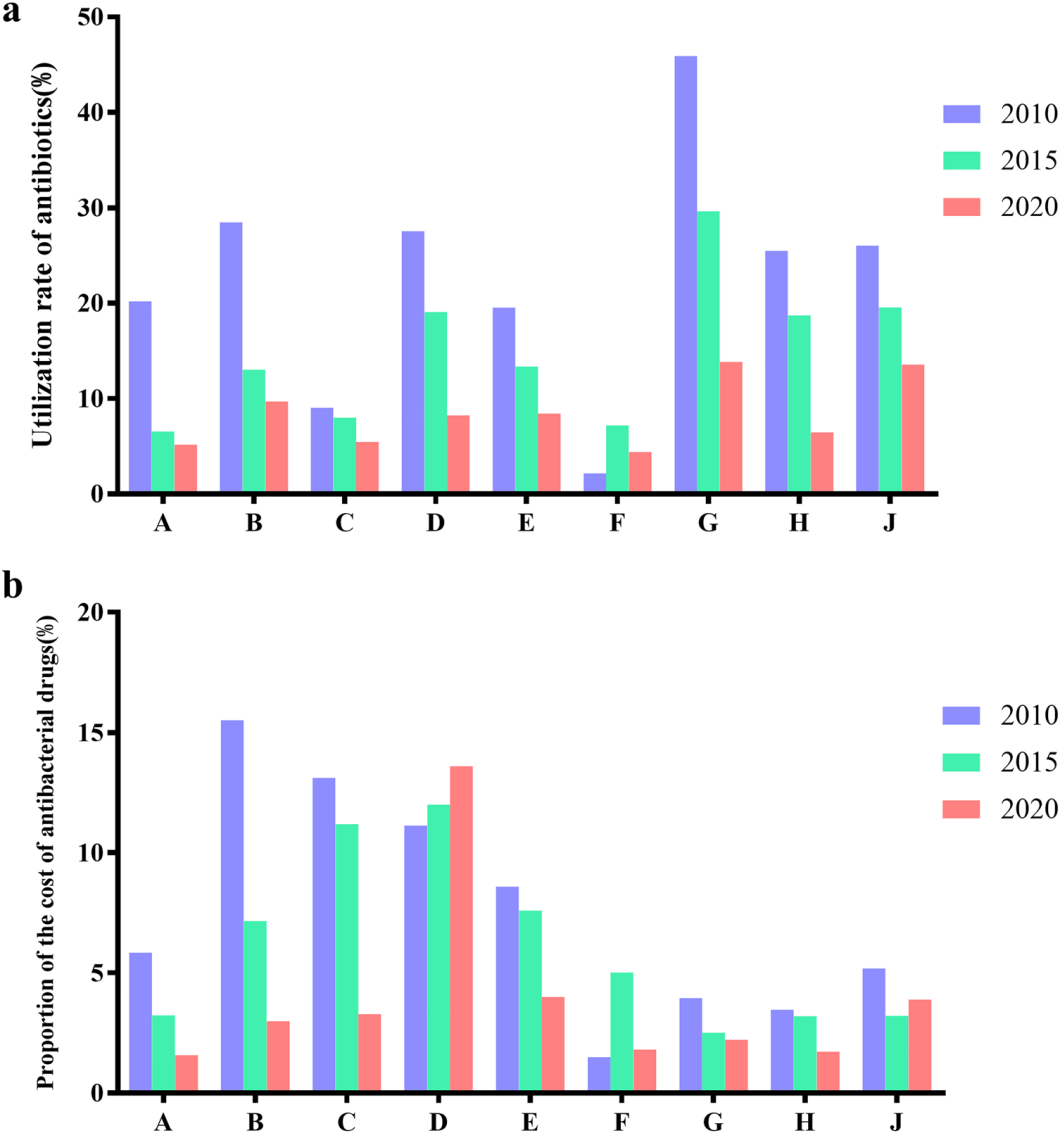
Usage of antibiotics in outpatient department of the nine hospitals in 2010, 2015 and 2020. (**a**) Utilization rate of antibiotics prescribed in outpatient department; (**b**) Proportion of antibacterial drug expenditure in outpatient department.

In 2010, the highest usage rate of outpatient antibiotics was 45.9%, which far exceeded the national standard (The utilization rate of antibiotics in outpatient is less than 20%), and only two hospitals met the standard. After rectification, in 2015, only one hospital had a utilization rate of antibiotics exceeding 20%, and other hospitals have met the standard. In the process of continuously implementing the SAC, the utilization rate of outpatient antibiotics was less than 20%, all of which meet the standard in 2020. The proportion of the total amount of antibiotics used in outpatient services also decreased significantly (Fig. 4). Compared with 2010, the usage rate of antibiotics and the percentage of antimicrobial drug costs in outpatient decreased significantly in 2020, indicating that the effect of SAC is obvious.

### Use of antibiotics in inpatient department of 9 hospitals

**Figure 5:**
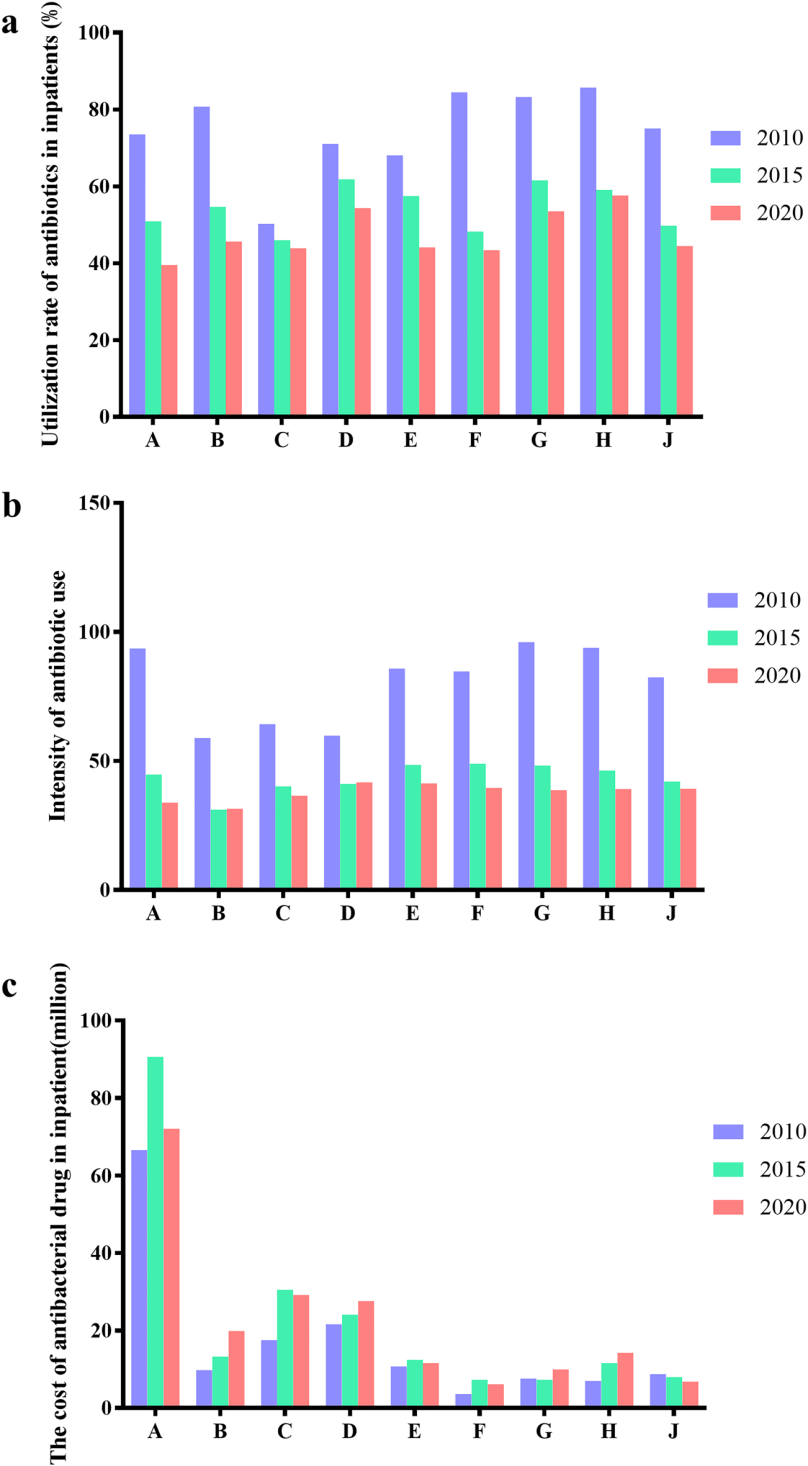
Usage of antibiotics in inpatient department of the nine hospitals in 2010, 2015 and 2020. (**a**) Utilization rate of antibiotics prescribed in inpatients; (**b**) Intensity of antibiotic use in hospitalized patients; (**c**) the cost of antibacterial drug in inpatient department.

According to the relevant indicators of antibacterial drug management, the antibacterial drug use rate of inpatients shall not be higher than 60%. However, in 2010, the antibacterial drug use rate of inpatients in most hospitals was higher than 60%, and the highest was 85.72%. However, after rectification, in 2015, only two hospitals were higher than 60%, but they were very close to the target value of less than 60%. However, in 2020, the use rate of antibiotics in the selected 9hospitals was less than 60% (Fig. 5a). In terms of intensity of antibiotic use, in 2010, the highest utilization rate of antibiotics in hospitals was 96.01 DDD per 100 bed-days, far exceeding the national standard (the intensity of antibiotics in hospitalized patients did not exceed 40 DDD per 100 bed-days), and no hospital met the standard. After rectification, in 2015, although the intensity of antibiotic use of only one hospital was lower than 40, the decline of antibiotic use in other hospitals was obvious, while in 2020, the intensity of antibiotic use in only two hospitals was higher than 40 DDD per 100 bed-days (Fig. 5b). It can be seen from Fig. 5c that the cost of antibiotics in hospital A is much higher than that in other hospitals.

### Prophylactic use of antibiotics during perioperative period

**Figure 6:**
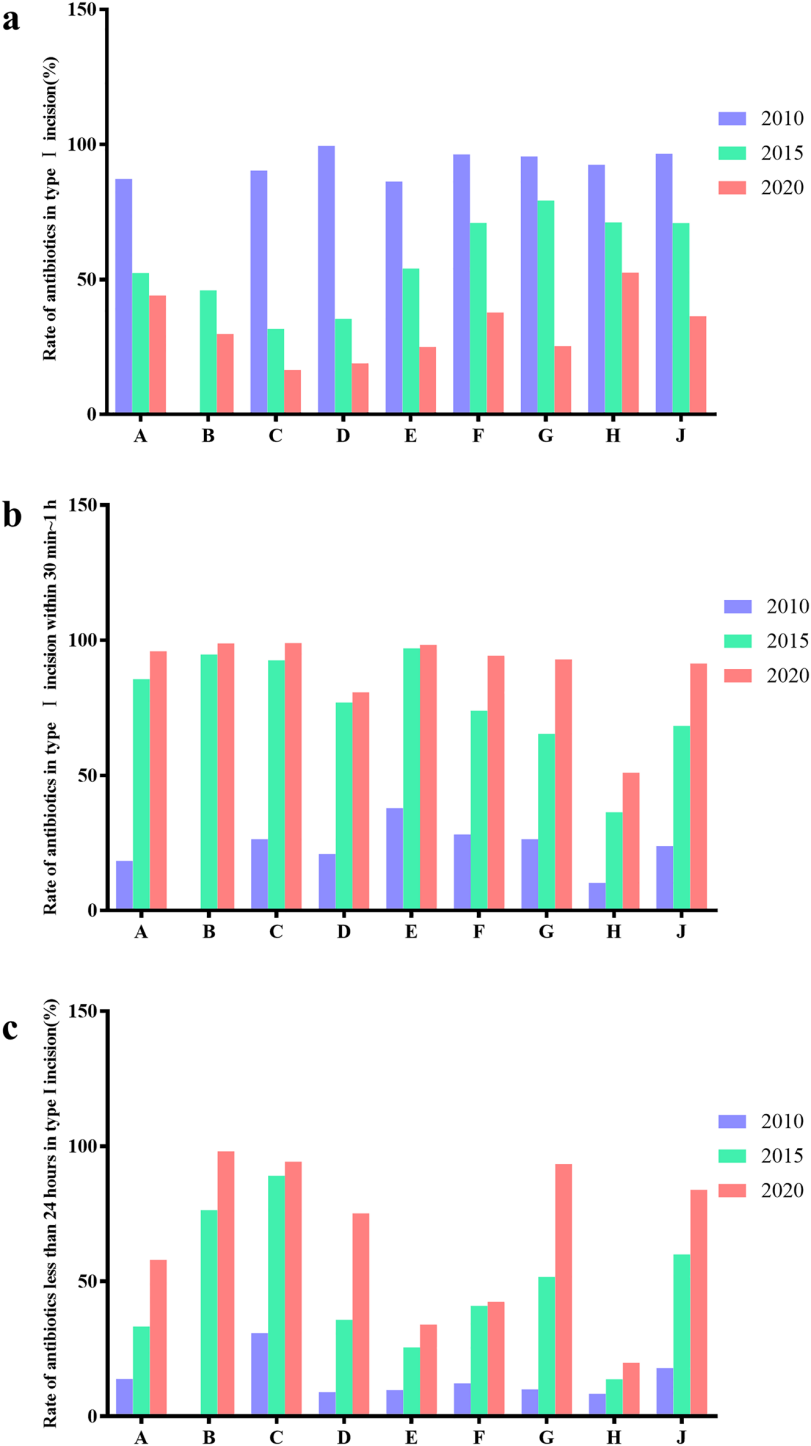
Usage of antibiotics in type I incision surgery of the nine hospitals in 2010, 2015 and 2020. (**a**) Utilization rate of antibiotics in type I incision surgery; (**b**) Utilization rate of antibiotics in type I incision operation within 30 min to 1 h before operation; (**c**) The proportion of prophylactic use of antibiotics less than 24 h in type I incision surgery.

The rational use of antibiotics during perioperative period can effectively prevent the occurrence of infection and the production of drug-resistant bacteria. However, in 2010, the use rate of perioperative antibiotics in selected 9 hospitals ranged between 80 and 99.5%, which is very amazing data. Fortunately, after rectification, the use of antibiotics during operations has decreased significantly. For example, the hospital C had decreased from the highest 99.48% in 2010 to 35.38% in 2015 and 18.96% in 2020 (Fig. 6a). In terms of the use time of antibiotics, the proportion of preventing the use of antibiotics within 30 min–1 h before operation was increasing. For example, in hospital A, the proportion of prophylactic use of antibiotics within 30 min–1 h before operation was only 18.36% in 2010, while in 2015 and 2020, the proportion reached 85.35% and 95.85%, respectively.

In terms of the time of using antibiotics, the first is to prevent the use of antibiotics, and the proportion of controlling the time of using antibiotics within 30 min–1 h before the operation is increasing. For example, in hospital A, only 18.36% of the time to prevent the use of antibiotics was controlled within 30 min–1 h before the operation in 2010, 85.35% in 2015, and 95.85% in 2020 (Fig. 6b). In terms of whether the time of prophylactic use of antibiotics exceeds 24 h, in 2010, the proportion of prophylactic use of antibiotics less than 24 h in most hospitals was low, and after rectification, the proportion increased. For example, the highest proportion in hospital B was 98.07% (Fig. 6c).

### The specific situation of antibiotics in the 9 hospitals in 2020

**Figure 7:**
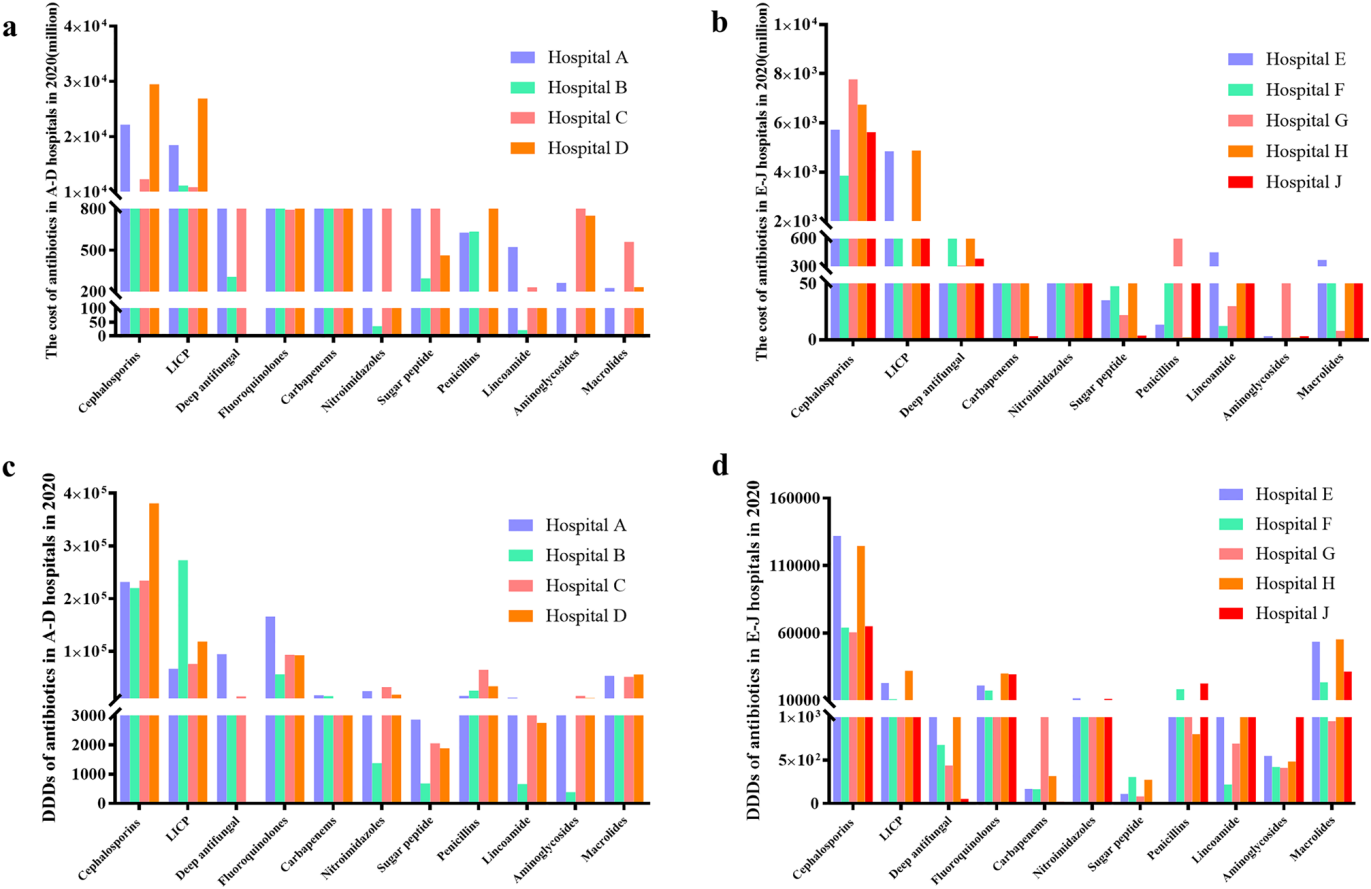
Usage of different antibiotics in different hospitals in 2020. (**a**) Total drug cost t of different antibiotics in A-D hospitals; (**b**) Total drug cost of different antibiotics in E-J hospitals; (**c**) DDDs of different antibiotics in A-D hospitals; (**d**) DDDs of different antibiotics in E-J hospitals. LICP: lactamase inhibitor compound preparation.

With the development of medical level and hospital scale, after 10 years, all 9? hospitals have been upgraded to tertiary hospitals, including four tertiary A hospitals and five tertiary B hospitals. We compared the consumption of money and DDDs of different types of antibiotics in these hospitals in 2020. The study found that although hospitals were different, the trend of drug use was the same. For example, cephalosporins were the most used in hospitals. However, β-Lactamase inhibitors were higher in tertiary A hospitals than in tertiary B hospitals (Fig. 7).

## Discussion

In this study, we summarized and analyzed the use of antibiotics in 9 hospitals in Southern Sichuan in the past 10 years, and provided long-term comparative data of antibiotis This enables us to analyze the use of antibiotics in Chinese hospital understand whether the SAC have achieved results, which will provide information for health care providers, decision makers and the public.

According to the number of outpatients and inpatients, we can roughly see the scale of the hospital. Therefore, it can be seen that hospital A is the largest scale, but the ratio of antibacterial drug cost to total drug cost is not the highest. On the contrary, whether in 2010 or 2020, the ratio of antibacterial drug cost is relatively low. This shows that the use of antibiotics is also relatively standardised in relatively large-scale hospitals, which is consistent with other reports: Kangkang Yan^18^ reported that antibiotic injection prescriptions accounted for 26.6% of all antibiotic prescriptions in secondary hospitals, while its share was only 14.2% in tertiary hospitals, within Shaanxi province in western China. In 2015 and 2020, the total number of antibiotics decreased compared with 2010, but in 2020, only the number of oral products increased. This also shows a signal that medical institutions have gradually formed a consensus and try to give oral preparations if they can take them orally. Studies have shown that the use of oral antibiotics for bone and joint infections is not inferior to intravenous injection^19^.

It can be seen from the above that hospital A is a comprehensive hospital. Figure 3 depicts the main antibiotics used during 10 years of the study. Cephalosporins and β-lactamase inhibitor compound preparation had a high use amount. Interestingly, there was a peak in the use of these two types of antibiotics in 2015, which may be due to the restrictions on the varieties of antibiotics in the first 5 years of the SAC. The use rate of outpatient and inpatient antibiotics showed a downward trend in 2010, 2015, and 2020, indicating the effect of SAC. At 2020, the use rate of antibiotics in hospitalized patients was basically maintained at about 40%. A tertiary care hospital in Beijing reported that use rate of antibiotics in inpatients was 34% in 2016^20^. However, the results of a cross-sectional survey of the nursing home cohort in the United States in 2017 showed that the use rate of antibiotics was 8.2%^21^. This shows that there is still a gap between the use of antimicrobial drugs in China and the US. The use intensity of antibiotics in hospitalized patients should not exceed 40 DDD per 100 bed-days, according to the Guidelines for the Clinical Use of Antimicrobial Drugs (hereinafter referred to as the Guidelines). It can be seen from Fig. 5b that before the SAC, no hospitals could meet the standard in 2010, far exceeding the value, indicating that the use of antibiotics may be seriously unreasonable. After SAC, in 2015, although the use intensity of antibiotics of some hospitals was still greater than 40 DDD per 100 bed-days, it decreased significantly. In 2020, the use intensity of antibiotics of most hospitals was lower than 40 DDD per 100 bed-days. This showed that the effect of SAC was remarkable. During process of SAC in China, the utilization rate of outpatient antibiotics and inpatient antibiotics decreased, which was consistent with that of other hospitals in China reported in 2015^22^.

There is certain risk of infection at the surgical site after the operation. There is a risk of infection at the surgical site after surgery. A variety of perioperative interventions can help reduce the incidence of Surgical Site Infection (SSI), and not all procedures require antibiotic prophylaxis^23^. According to the Guidelines, the perioperative prophylactic use rate of antibiotics in class I incision surgery should be below 30.0%. However, in 2010, it was more than 85%. This indicated that the perioperative abuse of antibiotics in class I incision surgery was very serious. After SAC, it improved, but still failed to meet the standard in 2015. In 2020, most hospitals had met the standard, with a minimum of 16.46%, but some hospitals, particularly hospital A, were still above 40%. The reason may be that hospital A is a comprehensive tertiary hospital, and most of the patients treated are difficult and severe, with difficult surgery and long time, or complicated with other chronic diseases, resulting in the need for preventive use of antibiotics in the perioperative period^24^. In terms of medication timing, the proportion of preventing the use of antibiotics within 30 min–1 h before surgery was very low in 2010. However, in 2020, it generally reached 80%, even more than 90%, and only one hospital was at 51.03%. This may be because the hospital G is located in a poor county in China and its medical resources are underdeveloped. An article reported the use of perioperative antibiotics in two Italian hospitals, the proportion of antibiotics administered within 30 min to 1 h before operation is also different in local and level hospitals^25^. For cesarean section, the one-time prophylactic application of antibiotics 30 min before operation can effectively prevent the infection after cesarean section and shorten the hospital stay^26^.

According to the analysis of the types of antibiotics used, it was found that cephalosporins were the most used antibiotics in all hospitals in 2020, similar to 26 hospitals in Saudi Arabia^27^. On the one hand, it may be due to the large number of varieties of cephalosporin, which now have one, two, three, four, and five generations. On the other hand, it has a wide antibacterial spectrum and relatively few side effects, which is a commonly used antibacterial drug for outpatients and inpatients^28^. Secondly, enzyme inhibitors and quinolones account for the largest proportion, which was consistent with the situation in Shanghai, China^29^. Carbapenems, glycopeptides and linezolid are mostly used only for the treatment of severe antibiotic-resistant infections and relatively infrequently for these drugs. Countries also have corresponding guidelines to guide the rational use of these antibiotics, such as the guidelines for vancomycin in the United States^30^, use and management of carbapenem antibiotics in Japan and Southeast Asian countries^31^.

However, there are also deficiencies in the article, and the situation of drug-resistant bacteria is not analyzed. The monitoring of bacterial drug resistance can also provide a strong basis for the clinical control of infection, the prevention of new drug-resistant strains, and the rational application of antibiotics. It will help to implement the situation of drug-resistant bacteria in the next step. Although after more than 10 years of development, various indicators of antibiotics in medical institutions in China have been greatly improved, there are still many problems, such as chaotic varieties of antibiotics in clinical application, high utilization rate and great differences between different regions, and severe challenges to the situation of bacterial drug resistance. The reasons for these problems are not only the management system and supervision ability of rational drug use, and the ability and level of rational drug use of medical personnel, but also the public awareness of rational drug use. It needs the joint efforts of the state, society, and medical personnel to jointly promote the rational use of antibiotics.

## Conclusion

After a long-term SAC to improve the clinical application of antibiotics, the rationality of the use of antibiotics in medical institutions at all levels in Southern Sichuan has been greatly improved. The use rate of antibiotics in outpatients and inpatients, perioperative preventive drugs, have been significantly improved. The clinical application management of antibiotics is related to the medical quality and the safety of patients. The hospital will continue to strengthen the training of medical personnel on the rational application of antibiotics and standardized management, further improve and refine the rules and regulationsand promote the rational application of antibiotics in the hospital.

## Data Availability

All data generated or analysed during this study are included in this published article.
